# Ovine Enzootic Abortion (OEA): a comparison of antibody responses in vaccinated and naturally-infected swiss sheep over a two year period

**DOI:** 10.1186/1746-6148-3-24

**Published:** 2007-09-28

**Authors:** Andrea Gerber, Ruedi Thoma, Evangelia Vretou, Evgenia Psarrou, Carmen Kaiser, Marcus G Doherr, Dieter R Zimmermann, Adam Polkinghorne, Andreas Pospischil, Nicole Borel

**Affiliations:** 1 Institute of Veterinary Pathology, Vetsuisse Faculty, University of Zurich, Switzerland; 2 Cantonal Laboratory of Veterinary Bacteriology, Chur, Switzerland; 3 Department of Microbiology, Hellenic Pasteur Institute, Athens, Greece; 4 Department of Clinical Veterinary Medicine, Vetsuisse Faculty, University of Berne, Switzerland; 5 Institute of Clinical Pathology, University Hospital, Zurich, Switzerland

## Abstract

**Background:**

Prevention and control of ovine enzootic abortion (OEA) can be achieved by application of a live vaccine. In this study, five sheep flocks with different vaccination and infection status were serologically tested using a competitive enzyme-linked immunosorbent assay (cELISA) specific for *Chlamydophila (Cp.) abortus *over a two-year time period.

**Results:**

Sheep in Flock A with recent OEA history had high antibody values after vaccination similar to Flock C with natural *Cp. abortus *infections. In contrast, OEA serology negative sheep (Flock E) showed individual animal-specific immunoreactions after vaccination. Antibody levels of vaccinated ewes in Flock B ranged from negative to positive two and three years after vaccination, respectively. Positive antibody values in the negative control Flock D (without OEA or vaccination) are probably due to asymptomatic intestinal infections with *Cp. abortus*. Excretion of the attenuated strain of *Cp. abortus *used in the live vaccine through the eye was not observed in vaccinated animals of Flock E.

**Conclusion:**

The findings of our study indicate that, using serology, no distinction can be made between vaccinated and naturally infected sheep. As a result, confirmation of a negative OEA status in vaccinated animals by serology cannot be determined.

## Background

*Chlamydophila abortus *(formerly Chlamydia psittaci serotype 1) is the most common infectious bacteria causing abortion in small ruminants in Switzerland with a previous study demonstrating that 39% of the examined abortions in sheep and 23% in goats were caused by this agent [1]. In the Swiss canton of Graubünden, a mountainous region in the countries' east, the economic losses associated with ovine enzootic abortion (OEA) are significantly higher than in other cantons [2].

*Cp. abortus *is generally introduced into immunologically naïve flocks by a latently infected animal with the agent being subsequently transmitted from aborting ewes via shedding of large amounts of infectious *Chlamydia *in the foetal membranes and in vaginal discharges [3]. In newly infected flocks, up to 30% of ewes may abort in the last trimester of gestation or give birth to weak or dead lambs. After abortion, ewes in these flocks may develop a protective immunity. Subsequent yearly losses in endemically infected flocks may decrease to a lower level (eg. 5–10%) with sheep either born into the flock or newly introduced animals likely to suffer abortions during their initial pregnancies [4,5].

Prevention and control of OEA is achieved by vaccination and/or treatment with oxytetracyclines [6]. Two vaccines against chlamydial abortion are licensed in Switzerland by the Federal Veterinary Office (FVO) in Berne. The first of these available was an egg-grown, formalin-inactivated, whole-organism vaccine (Ovax Clamidia, Fatro, Italy) which reduces the incidence of abortion in vaccinated herds but not completely [7-10]. Since December 2002, an avirulent, temperature-sensitive, live chlamydia vaccine (Ovilis^®^Enzovax, Intervet, The Netherlands), which is marketed to induce strong long-lasting protection, has been made commercially available in Switzerland. The attenuated strain 1B, which forms the basis of this vaccine, was obtained from the virulent *Cp. abortus *strain AB7 by nitrosoguanidine mutagenesis [11-13].

In 2005, a small pilot study was undertaken to determine if administration of vaccines to protect sheep flocks from OEA would result in antibody levels in the complement-fixation test (CFT) and in the competitive enzyme-linked immunosorbent assay (cELISA) tests similar to those following natural infection [14]. After vaccination with the inactivated vaccine (Ovax Clamidia) only one sheep developed a detectable antibody response. In contrast, vaccination with the attenuated live vaccine (Ovilis^®^Enzovax) resulted in detectable antibody titers in all tested sheep.

The aim of this study is to investigate a larger number of sheep over a two-year period in the field to compare flock-level ELISA responses between (a) vaccinated (live vaccine), (b) naturally infected and (c) non-infected sheep flocks. It was anticipated that the follow up study of the humoral responses could possibly discriminate between vaccinated and naturally OEA-infected sheep. An additional objective of the study was to attempt to detect chlamydiae and/or the attenuated strain of *Cp. abortus *used in the live vaccine in conjunctival swabs of sheep.

## Results

### Serological results and abortion cases

**Figure 1:**
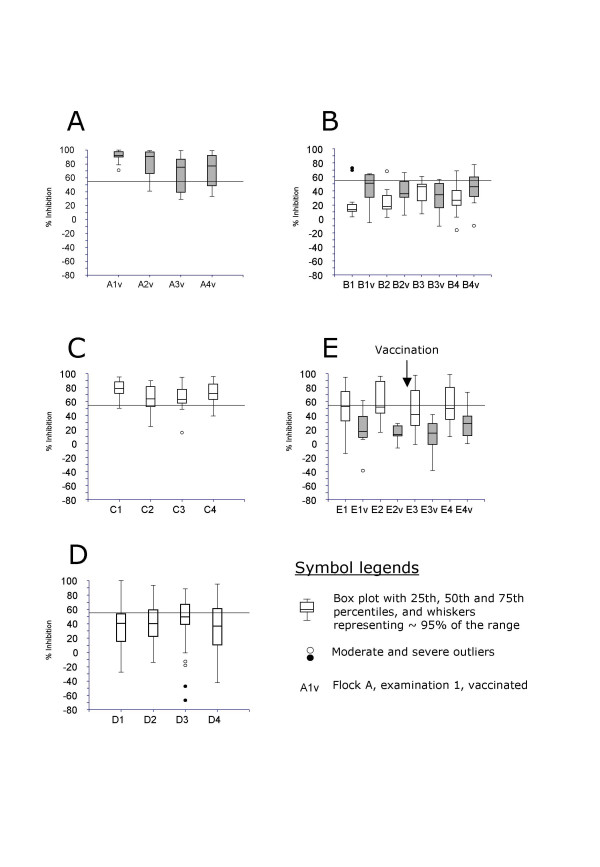
**Box plots of cELISA antibody values of all examined sheep over the four investigation dates**. Some or all animals in flocks A, B and E were vaccinated at given times (gray boxes).

cELISA classifications (frequency and proportion positive), median titer and respective range of positive classified sheep in flocks A, B, C, D and E over the four different investigation dates are shown in Table 1. The comparison between vaccinated and non-vaccinated animals in Flock B and E is shown in Table 2. Figure 1 shows the titer ranges (box plots) of all examined sheep in the five flocks over the four investigation dates.

**Table 1:** Serological results A, B, C, D and E. cELISA positive (above cutoff) sheep with frequency, respective proportion (%), median titers and titer range.

Flock (n)	Parameter	Spring 2005	Autumn 2005	Spring 2006	Autum 2006
A	No. positive	15	13	11	11
15 sheep	Prop. Pos. (%)	100	87	73	73
	Median titer	91.7	86.6	81.3	82.3
	Titer range	70.9 – 99.9	55.1 – 99.2	62.0 – 99.0	55.3 – 99.2
					
B	No. positive	8	4	2	8
26 sheep	Prop. Pos. (%)	31	16	8	31
	Median titer	65.0	62.9	58.7	63.9
	Titer range	61.3 – 72.8	55.1 – 68.1	56.4 – 61.0	55.3 – 77.5
					
C	No. positive	14	13	14	15
17 sheep	Prop. Pos. (%)^1^	82	76	82	88
	Median titer	82.9	72.2	71.3	76.8
	Titer range	69.1 – 95.2	55.1 – 89.6	57.1 – 94.8	55.4 – 95.8
					
D	No. positive	13	21	29	19
63 sheep	Prop. Pos. (%)	21	33	46	30
	Median titer	69.5	69.1	69.4	74.3
	Titer range	55.1 – 100	55.1 – 93.4	55.6 – 88.6	56.9 – 95.2
					
E	No. positive	26	24	19	24
63 sheep	Prop. Pos. (%)	42	38	30	38
	Median titer	73.4	81.2	80.4	81.8
	Titer range	57.4 – 94.8	56.5 – 96.3	55.2 – 97.6	56.0 – 98.7

^1^significant difference in % positive (Fishers Exact Test, p = 0.024)

**Table 2:** Serological results vaccinated vs. non-vaccinated (Flock B and E). Comparison of cELISA positive (above cutoff) vaccinated and naturally exposed sheep with frequency, respective proportion (%), median titers and titer range.

Flock (n)	Parameter	Spring 2005	Autumn 2005	Spring 2006	Autum 2006
B^1^	No. positive	6	3	2	2
14 sheep	Prop. Pos. (%)	43	22	14	43
	Median titer	62.9	61.3	58.7	64.5
	Titer range	61.3 – 64.2	55.1 – 66.3	56.4 – 61.0	55.3 – 77.5
					
B^2^	No. positive	2	1	0	2
12 sheep	Prop. Pos. (%)	17	8	0	17
	Median titer	71.1	68.1	-	62.2
	Titer range	69.4 – 72.8	-	-	55.9 – 68.5
					
E^1^	No. positive	1	0	0	1
13 sheep	Prop. Pos. (%)	8	0	0	8
	Median titer	61.4	-	-	73.2
	Titer range	-	-	-	-
					
E^2^	No. positive	25	24	19	23
50 sheep	Prop. Pos. (%)	50	48	38	46
	Median titer	76.3	81.2	80.4	82.2
	Titer range	57.4 – 94.7	56.5 – 96.3	55.2 – 97.6	56.0 – 98.7

^1^vaccinated group

^2^non-vaccinated group

All ewes (n = 15) of Flock A were serologically positive after vaccination showing a high median antibody value of 91.7%. The median antibody level of positive sheep (n = 13) decreased marginally to 86.6% in autumn 2005. In spring 2006 and autumn 2006, the seroprevalence in the flock was 73% (n = 11). The median antibody value of the positive sheep was 81.3% (spring 2006) and 82.3% (autumn 2006).

In spring 2005, two years after the first vaccination, six out of 14 vaccinated sheep in Flock B had a positive serological result (median antibody value 62.9%), whereas two out of 12 non-vaccinated sheep in the same flock were positive. The number of positive sheep decreased to three and two in the vaccinated group (n = 14) in autumn 2005 and spring 2006, respectively. In the non-vaccinated group, one sheep tested positive in autumn 2005, but none in spring 2006. In autumn 2006, the number of positive sheep increased in the vaccinated (n = 6) and non-vaccinated (n = 2) group, although abortions were not reported. The mean antibody values in the two groups were comparable, both being slightly greater than 60%.

Flock C (naturally infected flock) served as the positive control. The seroprevalence in sheep in spring 2005 was high at 82% (n = 14). The median antibody value in the positive group was 82.9%. The seroprevalence remained continuously high (76%–88%) during the whole study period and median antibody values in positive sheep were above 70%. In autumn 2005 newborn lambs were largely negative and had a significantly lower median antibody value than older ewes (Kruskal Wallis test, p < 0.05) (data not shown). The ewe with the confirmed chlamydial abortion in spring 2005 had positive antibody levels for the remaining sampling period comparable to the other animals in the flock (50.5%–77%). The seroprevalence in goats after confirmed chlamydial abortion in all four animals in spring 2005 was 100% (n = 4) with a high median antibody value of 91.6% (data not shown). In contrast to the sheep, all goats remained serologically positive with very high antibody values (71.2%–97.5%) over the whole testing period (data not shown).

Flock D served as the negative control for this study. Despite this, 21% (n = 13) of the ewes showed positive results in spring 2005, whereas 44% (n = 28) of the ewes had negative serological results and 35% (n = 22) of animals showed questionable readings. The median antibody values of the positive animals were 69.5%. Half a year later, in autumn 2005, 21 animals continued to be serologically positive. In spring 2006, the seroprevalence increased to 46%, whereas the mean antibody values of the positive animals were comparable to spring and autum 2005 (around 69%). In autumn 2006, the number of serologically positive ewes decreased to 30% (n = 19), whereas the mean antibody value of positive sheep increased to 74.3%.

Prior to vaccination in spring 2005, only one animal in Flock E was positive in the vaccination group (antibody value 61.4%), whereas 25 sheep (50%) were positive in the non-vaccinated group (n = 50). All 13 sheep of the vaccinated group were serologically negative in autumn 2005 and therefore selected for vaccination in winter 2005. The non-vaccinated group showed seroprevalences between 38–48% from autumn 2005 to 2006 and median antibody values of positive animals were consistently between 80.4–82.2%. In comparison to vaccinated sheep in Flock A, none of the animals vaccinated in winter 2005 were serologically positive in spring 2006. In autumn 2006, one ewe had a positive antibody value of 73.2%, whereas the other 12 vaccinated sheep had negative (n = 6) or questionable values (n = 6).

### Statistical comparison of mean titers

In flocks A (all animals vaccinated), C and D (no animals in both flocks vaccinated, Figure 1), differences in titer values between the sampling periods were always highly significant in the RM ANOVA model (p < 0.01). In Flock B, with a vaccination date between sampling periods 2 and 3, both vaccination status and an interaction term between vaccination and visit were statistically significant (p < 0.05). In Flock E, in which vaccination took place before the first sampling, both main effects were significant (time: p < 0.05; vaccination status: p < 0.01), while the interaction term was not.

### PCR results of eye swabs

In Flock E, 118 conjunctival swabs were collected before application of the live vaccine in autumn 2005. No obvious signs of ocular surface diseases such as conjunctivitis and keratitis were observed in any animal. IGS-S PCR screening detected 22 samples that were positive for chlamydial DNA. Sequencing of these PCR products identified 18 samples that shared greater than 98% sequence similarity to *Cp. abortus *[GenBank: CR848038.1]. One sample each was revealed to be positive for *Cp. pecorum *[GenBank: CPU68434] and *Cp. felis *[GenBank: AP006861.1]. The identity of two samples could not be determined.

Five months after vaccination, in spring 2006, 118 eye swabs were sampled in the same flock. 12 samples were tested positive by the IGS-S PCR but all were from non-vaccinated ewes. Of these samples, 5/12 were positive for *Cp. abortus *[GenBank: CR848038.1] while three were positive for *Cp. pecorum *[GenBank: CPU68434]. The identity of four samples could not be determined. None of the vaccinated sheep showed a positive IGS-S PCR result and it was concluded that no excretion of the vaccine strain had occurred.

## Discussion

This study represents the first longterm chlamydial serological study comparing vaccinated and non-vaccinated flocks in Switzerland. The investigations were undertaken in the canton Graubünden, where numerous chlamydial abortion cases in sheep were previously reported [1] and the highest seroprevalence (43%) for *Cp. abortus *in Swiss cantons was observed [2].

The results obtained from this study confirm the previous observations of the pilot study [14] that serology (cELISA) cannot be used to distinguish between sheep vaccinated with the live attenuated vaccine and naturally-infected sheep. The antibody value range in the recently vaccinated Flock A was comparable to Flock C in which acute infections of *Cp. abortus *occured at the same time. In Flock A, very high antibody levels (around 90%) were visible in every vaccinated sheep (n = 15), whereas antibody levels of sheep in the previous pilot study were somewhat lower (around 60%) 21 days post vaccination [14]. As chlamydial abortion was reported in Flock A in the past, sheep could have been already serologically positive before vaccination and the very high antibody levels could represent an overlay of both abortion and vaccine-associated antibody values. The mean antibody value of positive animals decreased in both flocks (A and C) from spring 2005 to spring 2006. A chlamydial abortion was diagnosed in one goat from Flock C in autumn 2006 explaining the increasing seroprevalence and antibody value in this group of animals at that time. The antibody values in the goats of Flock C after an acute infection with *Cp. abortus *were higher and persisted at a very high level (80 to 90%) over the observation period compared to the situation in sheep. No correlation with protection was seen however, as a chlamydial abortion occurred in a seropositive goat which had previously aborted. This observation was also made in other goat flocks in canton Graubünden (R. Thoma, personal communication). Goats treated with the live vaccine also aborted. In general, it is known that if *Chlamydiae *are introduced in a naive flock, the losses are much higher in goats (60%) than in sheep (30%). The differences between goats and sheep are consistent with previous records and to date remain unexplained [15,16].

Antibody levels of vaccinated ewes of Flock B ranged from negative to positive two and three years after vaccination, respectively. Questionable antibody levels are either attributed to undiagnosed *Cp. pecorum *infections [17] or are possibly due to the vaccination in spring 2003. In a similar situation to the naturally infected sheep (Flock C), a slow decrease of antibody values was observed over the sampling period. This observation strongly suggests that serology (cELISA) cannot be used to distinguish between sheep vaccinated with the live attenuated vaccine and naturally-infected sheep as anticipated in the previous pilot study [14]. As a direct consequence to this, the confirmation of negative OEA status in vaccinated animals by serology cannot be made. This is unfortunate as reliable confirmation is important if an abatement of OEA through assembly of OEA-free flocks is to be performed as undertaken by the Sheep and Goat Health Schemes in England and Wales and the Premium Health Scheme in Scotland.

Positive antibody values have been observed in the negative control flock (Flock D), which had not been vaccinated and was free from chlamydial abortion. An explanation for the observations of an increasing antibody value amongst this flock is that the animals may have asymptomatic intestinal infections with *Cp. abortus *as presumed in previous studies [17,19]. An alternative scenario is that the ewes were infected with a less virulent strain of *Cp. abortus*, which provokes seroconversion but no abortion [17,20]. Fluctuations in the antibody levels could be the result of bacterial shedding during oestrus which provokes an induction of antibody levels without causing abortion [21,22]. Unfortunately, little is still known at this time about the ability of *Cp. abortus *to persist in animals (and the anatomical location of this persistent infection) compared to other chlamydial species, which require more investigations.

In Flock E, the serological reaction of 13 selected vaccinated sheep and the 50 non-vaccinated sheep in the flock was evaluated. Surprisingly and in contrast to the observations in the previous pilot study [14] and in the two vaccinated flocks A and B, six of 13 vaccinated sheep of Flock E showed no seroconversion eight months after vaccination. Only one ewe had a positive serological result (73.2%), comparable to the vaccinated sheep of Flock A and the naturally OEA-infected sheep of Flock C. The remaining six ewes had questionable antibody levels. The primary difference between animals in flocks A and E was the high variability of antibody levels in vaccinated animals. These results suggest that individual immunoreactions between sheep can vary considerably.

Sampling of conjunctival swabs from sheep in Flock E was performed to detect and compare the presence of chlamydial DNA before and after vaccination. Furthermore, a possible excretion of the vaccine through the eye could be screened with this approach. Although chlamydiae were frequently detected by PCR in conjunctival swabs of sheep, the attenuated strain of *Cp. abortus *used in the live vaccine was not detected in swabs collected from vaccinated sheep. The incidence of *Cp. abortus *and *Cp. pecorum *and even *C. suis *in clinically healthy non-vaccinated sheep was previously observed in a recent study [23]. The significance of this possible new mode of transmission for OEA needs further investigation.

## Conclusion

The findings in our study strongly suggest that serology (cELISA) cannot be used to distinguish between sheep vaccinated with the live attenuated vaccine and naturally-infected sheep. The course of antibody levels, nevertheless, can vary between individual animals and flocks. Compared to sheep, goats displayed higher antibody levels, which persist over a longer time period but do not correlate with protection. The attenuated strain of *Cp. abortus *used in the live vaccine was not detected in eye swabs collected from vaccinated sheep.

## Methods

### Flock details

Five different sheep flocks in the canton Graubünden were followed over a two-year period with four flock visits. These five flocks were available for the study in spring 2005 through an established collaboration with veterinary authorities in the canton Graubunden.

Due to constant turnover in each flock (i.e. slaughtering of old or sick ewes, birth of lambs, introduction of new animals) the number of animals tested all four times was much lower than the number of individual sheep in the flock. Details on the five tested flocks (A, B, C, D and E) over the four investigation dates (spring 2005/06, autumn 2005/06) are provided in Table 3. Briefly, animals of Flock A were available for serological testing after vaccination of 15 sheep in spring 2005. History of chlamydial abortion in autumn 2004 was reported, but none of the 15 sheep in the study suffered an abortion during the examination period. Ewes (n = 14) of Flock B were vaccinated in spring 2003 with the live vaccine because of a chlamydial abortion outbreak in the vicinity of this flock. Before and after vaccination, no abortions due to *Cp. abortus *occurred and, as a result, the owner abandoned a vaccination booster two years later. Access to this flock was possible in spring 2005. Flock C had an average of 11 goats over the four investigation dates, of which four were available for repeated testing during the four sampling periods, but the results were not included in the overall statistical calculations of Flock C. Flock C had confirmed chlamydial abortions in autumn 2004 (unknown number of animals) and spring 2005 (one ewe and four goats). No further chlamydial abortions occurred in this flock after spring 2005. Animals suffering from abortions were tested four times during the study. Flock D represented the negative control flock as no abortion or vaccination occured during the study period. Sheep in this flock spent summer together with a flock that had reports of chlamydial abortion in the past. Nevertheless, no abortions in Flock D were observed during that time. The ewes from Flock E (n = 63) suffered from chlamydial abortions for many years. The last confirmed case of chlamydial abortion was documented in spring 2005. In this flock, selected sheep (n = 13) that were negative by a cELISA screen in autumn 2005 were vaccinated with the live vaccine according to the instructions of the manufacturer in winter 2005 and tested two times after vaccination (spring/autumn 2006). The serology of the non-vaccinated sheep in this flock (n = 50) was also followed. During the investigations, no abortion due to *Cp. abortus *was observed in this flock.

**Table 3:** Flock details

Flock	Examination dates	Average no. sheep	Sheep tested all 4 times	Flock history	OEA status^1^	Vaccination with live vaccine
A	spring & autumn 2005/2006	54	15	chlamydial abortions in autumn 2004	positive	15 sheep (spring 2005)
B	spring & autumn 2005/2006	48	26	chlamydial abortion outbreak nearby in 2003	negative	14 sheep (spring 2003), no vaccination booster
C	spring & autumn 2005/2006	45^2^	17^3^	chlamydial abortions (positive control)	positive	no
D	spring & autumn 2005/2006	105	63	no abortions (negative control)	negative	no
E	spring & autumn 2005/2006	118	63	chlamydial abortions in the past	positive	13 sheep (winter 2005)

^1^OEA = ovine enzootic abortion

^2^Average no. goats: 11

^3^Goats tested all 4 times: 4

Blood samples were collected from each flock during spring and autumn of 2005 and 2006 using Vacutainer tubes Becton Dickinson, Heidelberg, Germany). Four hours after collection, blood samples were centrifuged at 3000 × g for 10 minutes and stored in Nunc CryoTubes (Nalge Nunc International, Roskilde, Denmark) at -20°C until further processing.

### cELISA

Serum samples were tested by the competitive enzyme-linked immunosorbent assay (cELISA) using the monoclonal antibody mAb 188 directed against the variable segments 1 (VS1) and 2 (VS2) of the major outer membrane protein (MOMP) of *Cp. abortus*, according to the protocol of Salti-Montesanto et al. [17]. The results of the cELISA were expressed as 'percentage of inhibition' corresponding to the antibody concentration in the sample. Inhibition values above 55 per cent were considered positive for infection with *Cp. abortus *(positive cut-off) whereas inhibition values between 30 – 55 per cent were classified as questionable, attributable to either *Cp. abortus *or *Cp. pecorum*, a widely distributed chlamydial agent in small ruminants causing diseases such as arthritis/conjunctivitis and pneumonia syndrome in lambs and also subclinical intestinal infections [18,19]. Inhibition values below 30 per cent were assumed to be negative [17,24].

### PCR of eye swabs

Conjunctival swabs (Cytobrushes, Berdat Charles, Bourroux, Switzerland) were collected from Flock E before and after vaccination to investigate possible excretion of chlamydiae and/or the *Cp. abortus *vaccine strain through the eye. Before application of the vaccine, conjunctival swabs from every sheep in the flock (n = 118) were collected in autumn 2005. Five months following vaccination (spring 2006), the second conjunctival swab samples were taken from every sheep in the flock (n = 118). Cytobrushes were each placed in a 1.5-ml Eppendorf tube and stored at -80°C until further processing. DNA extraction from all swabs was performed as described previously [25] using a commercial DNA extraction kit (DNeasy Tissue Kit^®^, Qiagen, Hombrechtikon, Switzerland).

The conjunctival swabs were investigated for the presence of chlamydial DNA by a *Chlamydiales*-order specific PCR targeting the intergenic spacer region (IGS) between chlamydial 16S and the 23S rRNA genes [26] and using primers cIGS1f (5'-CAA GGT GAG GCT GAT GAC-3') and cIGS2r (5'-TCG CCT KTC AAT GCC AAG-3'). PCR conditions are described elsewhere [26]. The identity of all positively tested IGS PCR products was determined by direct sequencing of the PCR product from both strands. Sequencing was performed with an ABI Prism 377 DNA sequencer (Applied Biosystems) or Applied Biosystems 3100 (Synergene Biotech). The obtained sequences were compared with the sequences available in GenBank using the BLAST server from the National Center for Biotechnology Information [27].

### Investigation of abortion cases

Abortion cases in the flocks were further investigated for the presence of chlamydiae by routine bacteriology and immunohistochemistry of the placenta and the fetal organs (lung, liver, kidney) as described elsewhere [28].

### Statistical analysis

Ewe ELISA antibody values were initially categorized into positive, questionable or negative as described previously [17,24]. For the analysis, questionable and negative results were both interpreted as negative. Whole flock response patterns over time were visualized using box plots. For those sheep that were tested all four times, the proportion of positive ewes at each time point was compared within each flock using a Fishers Exact Test with exact p-values. In addition, the mean titers of those sheep were compared using a repeated measures ANOVA with animal ID, time (within animal repetition factor), vaccination status (flocks B and E only), and the interaction between time and vaccination (again only for flocks B and E).

Data were stored and handled in MS Excel, and analysed using the statistical software packages NCSS 2004 [29] and SPSS 14 [30]. The overall level of statistical significance was set to 0.05.

## Competing interests

The author(s) declares that there are no competing interests.

## Authors' contributions

AG carried out the serum sampling and the serological investigations and drafted the manuscript. RT performed the investigation of the abortion cases and contacted the flock owners. EV and EP prepared the cELISA plates. CK investigated the eye swabs by PCR. MGD performed the statistical analysis. DRZ performed the DNA sequencing. AP assisted in the drafting and editing of the manuscript. APOS and NB participated in the design and coordination of the study. All authors read and approved the final manuscript.
